# Percutaneous thermal ablation combined with TACE versus TACE monotherapy in the treatment for liver cancer with hepatic vein tumor thrombus: A retrospective study

**DOI:** 10.1371/journal.pone.0201525

**Published:** 2018-07-31

**Authors:** Yang Wang, Liang Ma, Zhuhui Yuan, Jiasheng Zheng, Wei Li

**Affiliations:** 1 Center of Interventional Oncology and Liver Diseases, Beijing You’an Hospital, Capital Medical University, Beijing, China; 2 Cancer Center, Beijing Ditan Hospital, Capital Medical University, Beijing, China; Northwestern University Feinberg School of Medicine, UNITED STATES

## Abstract

**Purpose:**

To investigate the efficacy of percutaneous thermal ablation combined with transarterial chemoembolization (TACE) versus TACE monotherapy in treating primary liver cancer with hepatic vein tumor thrombus (HVTT), and to identify potential factors of overall survival after combination therapy.

**Materials and methods:**

Patients with primary liver cancer and HVTT from 2011 to 2016 at our institute were retrospectively identified. They were divided into two groups (group A and group B). Patients in group A underwent TACE with subsequent percutaneous thermal ablation, while patients in group B who were unsuitable for ablation received TACE monotherapy. Characteristics and survival data of the two groups were analyzed and compared. Relevant factors for overall survival (OS) of group A were explored by univariate analysis.

**Results:**

Twenty-six patients were included and analyzed. The median OS for group A (n = 13) was 18 months, while the 1-, 2- and 3-year survival rates were 58.6%, 46.9% and 46.9%, respectively. The median OS for group B (n = 13) was 6.5 months and the 1-year survival rate was 10.9%. The survival of group A was significantly better than group B (P = 0.02). The following factors were related with overall survival of group A: ablation technique, complete response of tumor and HVTT, Child-pugh grade, pre-operative extrahepatic metastases and lymph node metastases. In group A, patients who achieved complete response had the longest average survival time (42.1 months).

**Conclusion:**

For patients with primary liver cancer and HVTT, percutaneous thermal ablation and TACE present better efficacy than TACE monotherapy. Long-term survival could be achieved in selected patients.

## Introduction

Liver cancer is one of the most common malignancies in the world, with an estimated 782,500 new cases and 745,500 deaths worldwide in 2012[1]. The intrahepatic metastases usually spread via portal and hepatic veins (HV)[2]. The incidence of hepatic vein tumor thrombus (HVTT) is reported to be 4% in surgical and autopsy samples[3]. In most cases, HVTT presents as cordlike or columnar masses in the hepatic vein on computed tomography (CT) and magnetic resonance imaging (MRI). The tumor thrombus (TT) could show similar enhancement pattern as hepatocellular carcinoma (HCC), with hyper attenuation at the arterial phase and rapid washout at the portal venous phase[4]. HVTT usually originates from the tumor mass, while in some other cases the thrombus might be obliterated by the lesion on CT or MRI imaging [5].

For patients who are classified as BCLC advanced stage due to poor performance status or Child-Pugh grade, sorafenib might not be an option. More aggressive treatment could be considered after liver function and performance status are improved. For patients with HVTT, sorafenib is recommended [6]. But the prognosis remains poor, mainly due to the modest efficacy of sorafenib, rapid intrahepatic tumor progression, pulmonary metastases and secondary Budd-Chiari syndrome[7]. Moreover, the extension of tumor thrombus into the right atrium can cause intractable heart failure[7]. The median overall survival (OS) after sorafenib treatment is usually no more than one year[8], even combined with other therapies[9]. The high cost is a vital limitation of sorafenib therapy as well. Non-surgical treatments (including transarterial chemoembolization (TACE), hepatic intra-arterial infusion, stereotactic body radiation therapy, Y90 radioembolization, *etc*.) could be alternative therapies. However, few of these treatments prolong patient survival to one year or more[10, 11].

Radiofrequency ablation (RFA) and microwave ablation (MWA) are both thermal ablation techniques, and have been widely applied in treating liver cancer, with advantages of curative efficacy, minimal invasiveness and repeatability[12]. For RFA, the ablation area is easier to control due to the moderate heating process; thus it is less likely to injure adjacent organs[13]. On the other hand, MWA is capable of inducing larger ablation volume within shorter time duration, and is more resistant to the “heat-sink effect”[14]. TACE is often applied prior to ablation as a neoadjuvant therapy, since the chemotherapeutic agents and the embolization of tumor feeding arteries could enhance the therapeutic effect[15]. In addition, lipiodol could be applied as the vascular embolization agent, and its deposition in the tumor artery presents high density on CT imaging, which could help with the targeting process of CT-guided percutaneous ablation. Currently, the efficacy of thermal ablation combined with pre-operative TACE in treating liver cancer with HVTT has not been well studied yet.

This study aims to evaluate the combination therapy of percutaneous thermal ablation and TACE versus TACE monotherapy for patients with liver cancer and hepatic vein tumor thrombus, and to identify relevant factors for overall survival after the combination therapy.

## Materials and methods

### Patients

Patients with liver cancer and HVTT at our institute from 2011 to 2016 were consecutively enrolled. Inclusion criteria: I. Patients with primary liver cancer (including HCC and intrahepatic cholangiocarcinoma(ICC)); II. Concomitant HVTT was confirmed by pre-operative CT; III. Patient refused sorafenib therapy; IV. Eastern Cooperative Oncology Group performance status score < 3. Exclusion criteria: I. Patients with critical underlying disease including other types of cancer, solid organ transplantation history, immunosuppressive illnesses, diseases requiring continuous immunosuppressive agents treatment, uncontrolled organ (heart, lung, renal, liver, *etc*.) failure; II. Child-Pugh grade C; III. Patients with untreatable extrahepatic metastases.

Patients in group A underwent TACE and subsequent percutaneous thermal ablation. Patients in group B were unsuitable for ablation, and received TACE monotherapy. The following factors were considered unsuitable for ablation: I. Patients with too heavy tumor burden to be ablated; II. Tumor and HVTT were technically difficult for ablation; III. Patients with rapid tumor progression or deteriorating liver function; IV. Patients with severe coagulation disorders (platelet count less than 50×10^3^/μL or prothrombin activity less than 60%).

The patient and tumor characteristics were retrospectively collected. Written informed consent was obtained from each individual for their information to be stored and used for research. Human experimentation guidelines of China were followed. This study was approved by the institutional review board of our hospital ethics committee.

### Pre-operative TACE

Contrast-enhanced CT was performed before TACE for each patient, and was used as the reference imaging. TACE was performed under local anesthesia with 1% lidocaine. A 5-F pigtail catheter was introduced through the femoral artery, and the survey of tumor feeding arteries was performed. The chemotherapeutic agents, lipiodol (4–10 ml) and gelatin sponge particles (25–100 mg) were then sequentially injected under fluoroscopic guidance. Chemotherapeutic agents included the following: for HCC, oxaliplatin (100–200 mg) and fluorouracil (500 mg); for ICC, epirubicin (10 mg) and arsenic trioxide (10 mg). The dose of embolization agents was depending on the size and arterial supply of the tumor.

### Percutaneous thermal ablation

For patients in group A, thermal ablation was performed after TACE. Patients were under conscious sedation and local anesthesia. RFA (RITA Medical Systems, Mountain View, CA; Valleylab, ACTC1525, Boulder, Covidien; VIVA RF system, STARmed, Goyang, Korea; Celon AG medical instruments, Teltow, Germany) and MWA (Qinghai Ltd., Nanjing, P.R. China) were performed percutaneously under CT guidance (Toshiba, Tokyo, Japan). A 22-G puncture needle was used to lead the electrode/antenna to the target lesion. MWA was more frequently applied for large tumors adjacent to big vessels, and RFA was preferred to treat tumors with smaller size or near important tissues such as colon, heart or gallbladder. To ensure that the electrode/antenna was in the correct location, the angle and depth of each puncture were precisely calculated based on intraoperative CT scans. Ablative power and duration time were designed to induce a desired necrotic zone. After the procedure, the needle tract was ablated to prevent tumor dissemination and hemorrhage. Technical and ablation details (output power, temperature, time, *etc*.) for each patient were displayed in the supplemental material (**S1 Table**). The essentials for the ablation of HVTT were summarized in **S2 Table**.

### Complications

Complications of all procedures were assessed according to the Society of Interventional Radiology Clinical Practice Guidelines[16]. Complications were classified as major (complications requiring additional therapy, significantly prolonging hospital stay or leading to mortality/disability) and minor (complications resulting in no consequence, requiring no therapy or nominal therapy).

### Tumor response assessment

Tumor response was assessed by contrast-enhanced CT within one month after treatment. The CT was compared with the reference imaging obtained before TACE. Based on the Modified Response Evaluation Criteria In Solid Tumors (mRECIST)[17], tumor response was classified as complete response (CR), partial response (PR), stable disease (SD) and progressive disease (PD).

### Follow-up

Follow-up CT imaging was regularly obtained every 3 months. Image findings were retrospectively interpreted to evaluate technique efficacy and tumor. OS was defined as the time interval from the date of treatment to the date of death. Time to recurrence (TTR) of HVTT was defined as the time interval from the date of treatment to the date of tumor thrombus recurrence.

### Statistical analysis

Baseline differences between group A and group B were compared using SPSS 17.0 for Windows. Fisher's exact test was used to compare categorical variables, while Mann-Whitney U test was used for continuous data. At univariate analysis for OS of group A, Kaplan-Meier method and univariate Cox regression were performed for categorical variables and continuous variables, respectively. Statistical significance was defined as P value less than 0.05. Original database was included in the supplemental material (**S3 Table**).

## Results

### Patient and tumor characteristics

A total of 26 patients with liver cancer and HVTT were identified. Thirteen patients were included in group A and 13 patients were included in group B. For group A, there were 12 patients with HCC and 1 with ICC. There were 2, 5, 4 and 1 patients with tumor thrombus in the left, middle, right and accessory hepatic vein, respectively. Another patient presented with both left and middle HVTT. For group B, all patients suffered from HCC. There were no significant differences between group A and group B in terms of tumor number (median, 2 versus 4; P = 0.443), tumor diameter (median, 49 mm versus 38 mm; P = 0.789), length of TT (median, 43 mm versus 31 mm; P = 0.4) and other features. Five patients in group A underwent RFA, while 8 patients underwent MWA. RFA and MWA subgroups shared similar tumor characteristics in terms of tumor number (mean, 3 versus 2.6, P = 0.94) and length of HVTT (mean, 42.6 versus 52.9 mm, P = 0.56); but patients undergoing RFA had smaller tumors size (mean, 37.2 versus 65.9 mm, P = 0.03). The average time interval between TACE and ablation for group A was 10 days (range, 3–14 days). More detailed characteristics are listed in **Table 1**.

**Table 1:** Characteristics of group A and group B.

Variable	Group A (n = 13)	Group B (n = 13)	P of difference
Type of tumor			1
HCC	12	13	
ICC	1	0	
Gender			1
Male	9	9	
Female	4	4	
Pre-operative radiation			1
No	12	13	
Yes	1	0	
Liver cirrhosis			0.48
No	2	0	
Yes	11	13	
HBsAg			0.593
Negative	2	1	
Positive	10	12	
HCV-IgG			1
Negative	10	11	
Positive	1	0	
Pre-operative EHM			1
No	11	10	
Yes	2	3	
Pre-operative LNM			1
No	11	12	
Yes	2	1	
Location of HVTT			0.817
Left HV	2	4	
Middle HV	5	4	
Right HV	4	5	
Accessory HV	1	0	
Left + middle HV	1	0	
HVTT and tumor			1
TT within the tumor boundary	2	3	
TT beyond the tumor boundary	11	10	
TT in IVC			1
No	7	6	
Yes	6	7	
Child-pugh grade			0.16
A	12	8	
B	1	5	
Age, year	60, 31–73	58, 25–82	0.758
ALT, U/L	30.3, 15.5–278	55.8, 12.6–110.6	0.663
TBil, μmol/L	14.5, 9.6–37.5	22.1, 8.2–82.9	0.191
ALB, g/L	39.3, 28.9–45.3	36.8, 30.9–43.6	0.248
ALP, U/L	96.4, 69.8–459.7	88.8, 56–271.9	0.555
PLT, ~10^3^/μL	141, 102–398	123, 30–301	0.209
PTA, percent	98%, 67%-1.07%	85%, 54%-1.09%	0.257
AFP, μg/L	60.6, 7.56–74506	1928, 2.4–37271	0.118
CEA, μg/L	2.4, 0.4–6.9	2.3, 0.8–10.7	0.951
CA19-9, μg/L	30.5, 11.1–100	24.2, 7.8–120.3	0.535
Number of tumor	2, 1–6	4, 1–10	0.372
Diameter of tumor, mm	49, 25–108	38, 15–120	0.837
Length of TT, mm	43, 27–85	31,16–90	0.4

Abbreviations: HCC, hepatocellular carcinoma; ICC, intrahepatic cholangiocarcinoma; HCV, hepatitis C virus; EHM, extrahepatic metastases; LNM, lymph node metastases; HVTT, hepatic vein tumor thrombus; HV, hepatic vein; TT, tumor thrombus; IVC, inferior vena cava; ALT, alanine aminotransferase; TBil, total bilirubin; ALB, albumin; ALP, alkaline phosphatase; PLT, platelet; PTA, prothrombin time activity; AFP, alpha-fetoprotein; CEA, carcinoembryonic antigen; CA19-9, carbohydrate antigen 19–9.

### Tumor response

**Fig 1:**
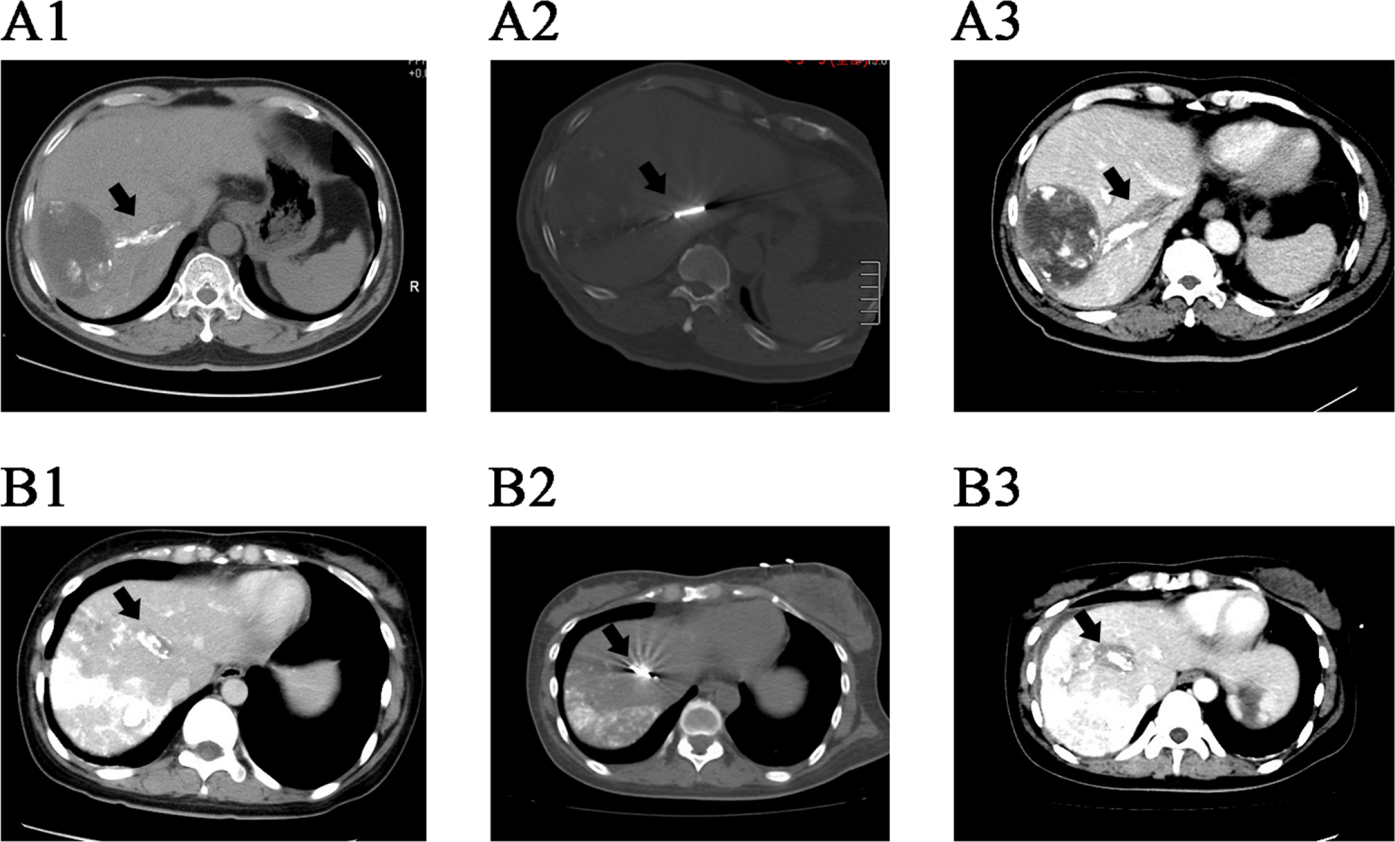
CT scanning of two patients with HVTT. (A1) CT scan of patient A after transcatheter arterial embolization showed tumor thrombus in the right hepatic vein. Lipiodol deposition in the thrombus feeding artery labeled the thrombus on CT imaging. (A2) Intra-operative CT scan of the ablation of thrombus. (A3) Instant post-operative contrast-enhanced CT of patient A showed complete necrosis of the thrombus: the thrombus was completely surrounded by the low density ablation zone. (B1) CT scan of patient B showed tumor thrombus and lipiodol deposition in the middle hepatic vein. (B2) Intra-operative CT scan of the ablation of thrombus. (B3) Instant post-operative contrast-enhanced CT of patient B showed that the thrombus was completely surrounded by the low density ablation zone. CT, computed tomography; HVTT, hepatic vein tumor thrombus.

For group A, HVTT showed complete necrosis on post-operative CT in 7 patients (53.8%). CR of both hepatic tumors and HVTT were observed in 6 of 13 patients (46.2%) in group A. The other patients were assessed as PR (7 of 13, 53.8%) after treatment. For group B, the PR rate was 92.3% (12 of 13); and another patient obtained SD (7.7%). In terms of complete TT necrosis, MWA induced 6 of 8 cases (75%), while RFA induced 1 of 5 cases (20%, P = 0.103). Imaging data of intra-operative ablation are exhibited in **Fig 1**.

### OS and recurrence analysis

**Fig 2:**
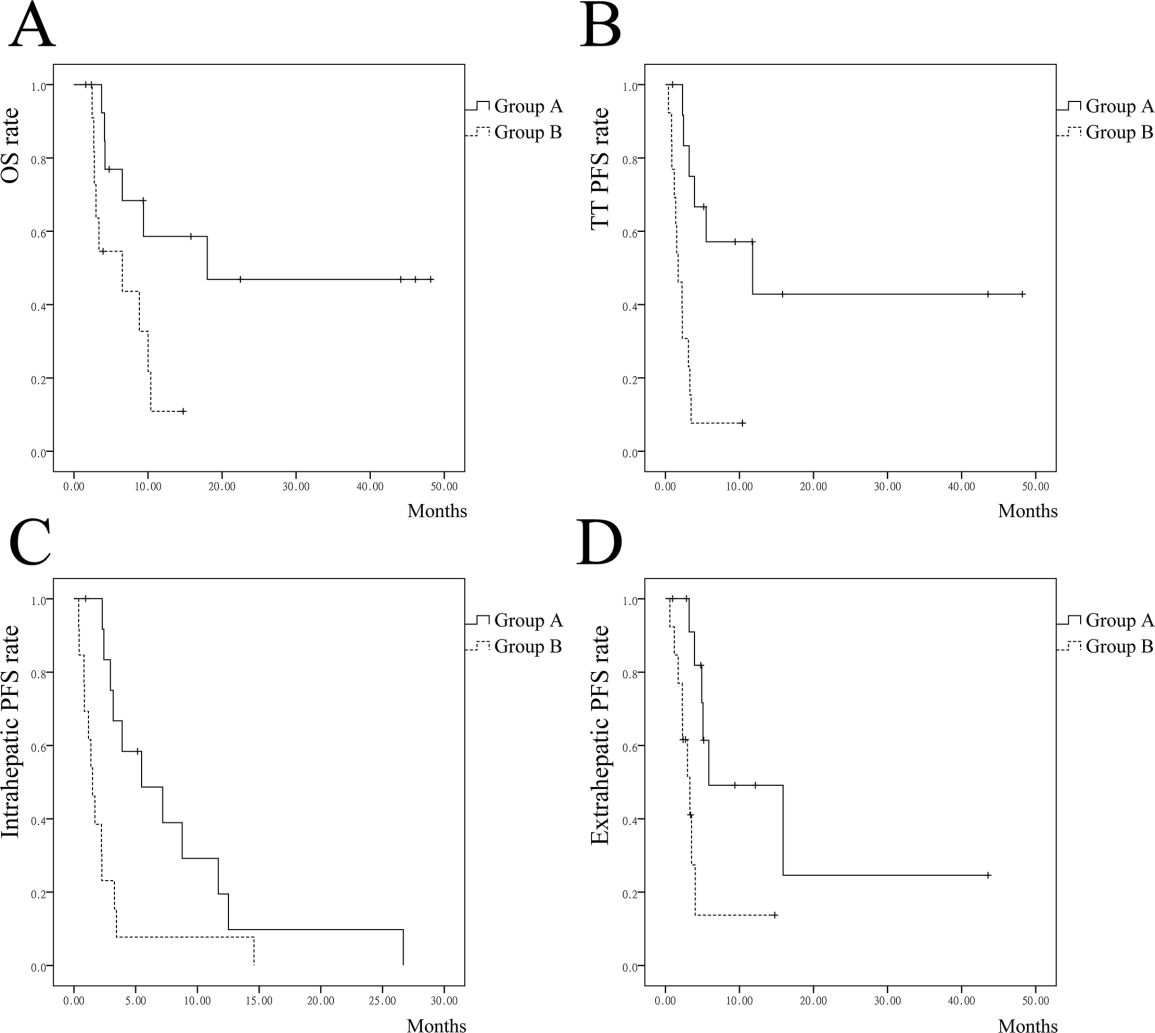
**Survival and recurrence of liver cancer patients with HVTT for group A and group B**. (**A**) Overall survival curves of group A and group B. (**B**) Tumor thrombus progression-free survival curves of group A and group B. (**C**) Intrahepatic tumor progression-free survival curves of group A and group B. (**D**) Extrahepatic metastases progression-free survival curves of group A and group B. HVTT, hepatic vein tumor thrombus; OS, overall survival; TT, tumor thrombus; PFS, progression-free survival.

Median follow-up duration was 29 months. Seven patients in group A survived at the end of follow-up. For group A, the median OS was 18 months and the 1-, 2-, 3-year OS rates were 58.6%, 46.9% and 46.9%, respectively. For HCC patients in group A (n = 12), the 1-, 2- and 3-year OS rates were 63.5%, 50.8% and 50.8%, respectively. Patient with ICC succumbed to disease within 4 months after treatment. Six patients who achieved CR had the longest average OS (42.1 months). Five of them survived at the end of follow up, while the other one succumbed to tumor progression and extrahepatic metastases 18 months after ablation. The other 7 patients in group A failed to achieve complete necrosis of tumor and HVTT, and average OS was 6.6 months. To further compare the patients who achieved CR versus others, no differences were found in terms of tumor number (mean, 2.5 versus 3), tumor diameter (mean, 63 versus 48 mm), extrahepatic and lymph node metastases (n = 0 versus 2), length of TT (mean, 48 versus 50mm) (P > 0.05, for all). But patients who achieved CR underwent more MWA procedures (6 of 6, versus 2 of 7, P = 0.021). For group B, the median OS was 6.5 months and the 1, 2, 3-year survival rates were 10.9%, NR (not reached), NR, respectively. Survival for group A was significantly better than group B (P = 0.02) (**Fig 2A**).

Median TTR of HVTT for group A was significantly longer than group B: 11.8 month versus 1.7 months (P < 0.001) (**Fig 2B**). Patients in group A also presented longer median time to intrahepatic recurrence (**Fig 2C**) and extrahepatic metastases (**Fig 2D**) than group B: 5.5 months versus 1.5 months (P = 0.005) and 5.8 months versus 3.3 months (P = 0.005), respectively.

### Univariate analysis of OS for group A

The following factors were statistically significant related with OS: ablation technique (P = 0.014), complete response of tumor and HVTT (P = 0.004), Child-pugh grade (P = 0.029), pre-operative extrahepatic metastases and lymph node metastases (P = 0.047) (**Table 2**). The size and number of intrahepatic tumors showed no relevance with OS (P > 0.05).

**Table 2:** Univariate analysis of OS for group A.

Variable	Median OS, months	P
Ablation technique		0.014
RFA	6.5*	
MWA	37.3*	
CR of tumor and HVTT		0.004
Yes	42.1*	
No	6.6*	
Child-pugh grade		0.029
A	29*	
B	4.1*	
EHM and LNM		0.047
Yes	6.6*	
No	31.6*	

Abbreviations: OS, overall survival; RFA, radiofrequency ablation; MWA, microwave ablation; CR, complete response; HVTT, hepatic vein tumor thrombus; TT, tumor thrombus; EHM, extrahepatic metastases; LNM, lymph node metastases.

*: median OS is not reached due to limited number of events, thus mean OS is presented instead.

### Subgroup analysis: Ablation + TACE versus TACE

**Fig 3:**
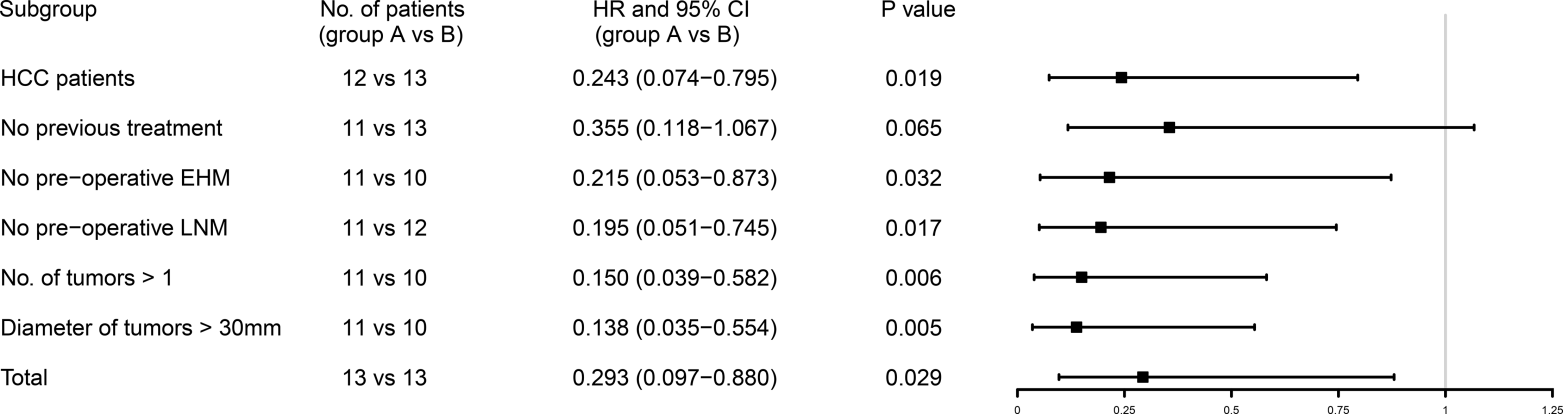
**Forest plot to show the hazard ratios for OS (group A versus group B) in selected subgroups.** HR, hazard ratio; CI, confidence interval; HCC, hepatocellular carcinoma; EHM, extrahepatic metastases; LNM, lymph node metastases.

The superiority between two therapies were further explored in selected subgroups (**Fig 3**). In most subgroups (including HCC patients, without pre-operative extrahepatic metastases or lymph node metastases, patients with multiple tumors and patients with tumors larger than 30 mm), the combination of ablation and TACE showed significantly better survival versus TACE alone (P < 0.05). Only in one subgroup (patients without previous treatment), the superiority of combination therapy was nearly significant (P = 0.065). Hazard ratio for OS was 0.293 (group A versus group B, P < 0.05).

### Complications

For TACE procedures, only minor complications were observed: 4 cases of slight fever, 2 cases of self-limiting pleural effusion and 1 case of hypertension. For ablation sessions, there was one major complication (biloma), which was managed by percutaneous transhepatic drainage. Minor complications for ablation included hepatic dysfunction (n = 4), hepatalgia (n = 4) and nausea (n = 2).

## Discussion

HVTT is different from liver cancer, due to its poor prognosis and intractable characteristics. According to the BCLC staging system, HCC patients with HVTT should be classified as advanced stage[6]. For untreated advanced-stage patients, estimated median survival was less than one year[6]. To date, there has been no consensus criteria in regard to the management of liver cancer with concomitant macroscopic vascular tumor thrombus involvement. Antitumor agents are recommended by the BCLC staging system[6]. Sorafenib is one of the most accepted antitumor agents, and has been proven to prolong patient survival for 3 months[8]. But few patients could get complete response or partial response through sorafenib, and median survival is usually less than one year[8].

Liver cancer with HVTT is a contraindication for surgical resection. Various non-surgical modalities have been attempted to treat HVTT, including hepatic artery chemotherapy and embolization[10, 18], radiotherapy[10], systemic drug therapy[10, 11, 19]. However, most previous studies were rare case reports, and other researches were limited by the sample scale and lack of controlled comparison. Most of the therapies could not prolong patient survival to more than one year[10, 11, 20, 21]. Combination therapy of TACE and ablation is usually used to treat liver cancer, especially early staged HCC, but rarely attempted for HVTT. In this study, the median survival time of TACE therapy (group B) was 6.5 months, similar to previous research. For HCC patients with vascular invasion who could not afford sorafenib, TACE might be an alternative therapy. The OS of patients undergoing ablation + TACE was more favorable than TACE (median OS: 18 versus 6.5 months, P < 0.05). Patients in group A also had better disease control of HVTT and intrahepatic tumors, as well as extrahepatic metastases (**Fig 2**). And further subgroup analysis showed that in most selected subgroups, thermal ablation combined with TACE also had better efficacy. The survival outcomes indicated a significant benefit of the combination therapy.

One patient had non-HCC tumor (ICC), and the survival result was unsatisfactory (less than 4 months of survival). ICC differed from HCC with more malignant characteristics. The patient had heavy hepatic tumor burden, as well as pulmonary metastases. These could lead to the poor survival outcome.

Complete ablation of tumor thrombus is of vital importance to the management of liver cancer patients with HVTT. In our study there were no significant differences of tumor number and the length of HVTT between patients undergoing RFA and MWA. The tumor size was smaller in patients undergoing RFA; however, MWA induced a 75% HVTT complete necrosis rate while RFA only induced 20% (P = 0.103). Furthermore, the survival outcome of patients undergoing MWA was significantly better than those undergoing RFA (**Table 2**), and these results might indicate that MWA appeared to be a better option for the management of HVTT.

MWA has been applied worldwide for its advantages such as its larger ablation zone, shorter duration, and resistance to the heat-sink effect[12, 22, 23]. Based on these characteristics, MWA is capable of reducing the frequency of puncture and maximizing the ablation zone, thus the risk of bleeding and ablation failure might be diminished, and the complete ablation rate of tumor thrombus could be improved. We suppose that these features of MWA contribute to its superiority upon RFA in treating tumor thrombus in the hepatic vein.

According to the survival factors analysis, selected patient groups are recommended for the combination therapy: HVTT patients without extrahepatic and lymph node metastases, patients with better liver function (Child-pugh grade, A) and patients with better chance of getting complete cancer response after treatment. For the latter, location and length of tumor thrombus are important, as well as tumor size and number. It is suggested that thermal ablation and TACE could be attempted for patients with TT easier to be ablated, smaller and less tumors. Long-term survival might be achieved for these selected patients.

There are several limitations of this study. First, TACE monotherapy was more preferred in patients with heavy tumor burden and rapid tumor progression, and there might be selection bias between group A and group B. We did comparison and found no baseline difference of two groups, and subgroup analysis also indicated that the survival in group A was better than that in group B in most subgroups. Thus the influence of selection bias might not be significant. Further randomized trials are warranted to eliminate the selection bias. Second, the differences among ICC and HCC were not compared, due to the limited amount of ICC patient. Third, this study is a retrospective research with limited number of patients, and the efficacy of thermal ablation and TACE need to be further validated in future multi-center randomized controlled studies.

## Conclusion

The combination of thermal ablation and TACE represents a useful and promising therapeutic modality for liver cancer patients with HVTT. Long-term survival could be achieved for selected group of patients. The efficacy of thermal ablation and TACE needs to be further validated in large-scale controlled trials.

## Supporting information

S1 Table Details of HVTT ablation procedures for each patient. (DOCX)

S2 Table Essentials for the ablation of HVTT. (DOCX)

S3 Table Database includes the original data for statistical analysis. (XLSX)

## Abbreviations

HV: hepatic vein
HVTT: hepatic vein tumor thrombus
CT: computed tomography
MRI: magnetic resonance imaging
TT: tumor thrombus
HCC: hepatocellular carcinoma
BCLC: Barcelona Clinic Liver Cancer
OS: overall survival
TACE: transarterial chemoembolization
RFA: radiofrequency ablation
MWA: microwave ablation
ICC: intrahepatic cholangiocarcinoma
mRECIST: Modified Response Evaluation Criteria In Solid Tumors
CR: complete response
PR: partial response
SD: stable disease
PD: progressive disease
TTR: time-to-recurrence
NR: not reached

## References

[1] Torre LA, Bray F, Siegel RL, Ferlay J, Lortet-Tieulent J, Jemal A. Global cancer statistics, 2012. CA: a cancer journal for clinicians. 2015;65(2):87–108. 10.3322/caac.21262 . 25651787

[2] Fong Y, Sun RL, Jarnagin W, Blumgart LH. An analysis of 412 cases of hepatocellular carcinoma at a Western center. Annals of surgery. 1999;229(6):790–9; discussion 9–800. ; PubMed Central PMCID: PMC1420825. 10363892 10.1097/00000658-199906000-00005 PMC1420825

[3] Tsuzuki T, Ogata Y, Iida S, Shimazu M. Hepatic resection in 125 patients. Archives of surgery. 1984;119(9):1025–32. . 6089699 10.1001/archsurg.1984.01390210029008

[4] Namasivayam S, Salman K, Mittal PK, Martin D, Small WC. Hypervascular hepatic focal lesions: spectrum of imaging features. Current problems in diagnostic radiology. 2007;36(3):107–23. 10.1067/j.cpradiol.2006.12.004 . 17484954

[5] Wei XB, Xu J, Li N, Yu Y, Shi J, Guo WX, et al The role of three-dimensional imaging in optimizing diagnosis, classification and surgical treatment of hepatocellular carcinoma with portal vein tumor thrombus. HPB: the official journal of the International Hepato Pancreato Biliary Association. 2016;18(3):287–95. 10.1016/j.hpb.2015.10.007 ; PubMed Central PMCID: PMC4814596. 27017169 PMC4814596

[6] Llovet JM, Bru C, Bruix J. Prognosis of hepatocellular carcinoma: the BCLC staging classification. Seminars in liver disease. 1999;19(3):329–38. 10.1055/s-2007-1007122 . 10518312

[7] Okada S. How to manage hepatic vein tumour thrombus in hepatocellular carcinoma. Journal of gastroenterology and hepatology. 2000;15(4):346–8. . 10824876 10.1046/j.1440-1746.2000.02151.x

[8] Llovet JM, Ricci S, Mazzaferro V, Hilgard P, Gane E, Blanc JF, et al Sorafenib in advanced hepatocellular carcinoma. The New England journal of medicine. 2008;359(4):378–90. 10.1056/NEJMoa0708857 . 18650514

[9] Zhang X, Wang K, Wang M, Yang G, Ye X, Wu M, et al Transarterial chemoembolization (TACE) combined with sorafenib versus TACE for hepatocellular carcinoma with portal vein tumor thrombus: a systematic review and meta-analysis. Oncotarget. 2017;8(17):29416–27. 10.18632/oncotarget.15075 ; PubMed Central PMCID: PMC5438741. 28177886 PMC5438741

[10] Murakami E, Aikata H, Miyaki D, Nagaoki Y, Katamura Y, Kawaoka T, et al Hepatic arterial infusion chemotherapy using 5-fluorouracil and systemic interferon-alpha for advanced hepatocellular carcinoma in combination with or without three-dimensional conformal radiotherapy to venous tumor thrombosis in hepatic vein or inferior vena cava. Hepatology research: the official journal of the Japan Society of Hepatology. 2012;42(5):442–53. 10.1111/j.1872-034X.2011.00943.x . 22176468

[11] Zhang YF, Wei W, Wang JH, Xu L, Jian PE, Xiao CZ, et al Transarterial chemoembolization combined with sorafenib for the treatment of hepatocellular carcinoma with hepatic vein tumor thrombus. OncoTargets and therapy. 2016;9:4239–46. 10.2147/OTT.S106659 ; PubMed Central PMCID: PMC4948732. 27471398 PMC4948732

[12] Kim YS, Lim HK, Rhim H, Lee MW. Ablation of hepatocellular carcinoma. Best practice & research Clinical gastroenterology. 2014;28(5):897–908. 10.1016/j.bpg.2014.08.011 . 25260316

[13] Poulou LS, Botsa E, Thanou I, Ziakas PD, Thanos L. Percutaneous microwave ablation vs radiofrequency ablation in the treatment of hepatocellular carcinoma. World journal of hepatology. 2015;7(8):1054–63. 10.4254/wjh.v7.i8.1054 ; PubMed Central PMCID: PMC4450182. 26052394 PMC4450182

[14] Poch FG, Rieder C, Ballhausen H, Knappe V, Ritz JP, Gemeinhardt O, et al The vascular cooling effect in hepatic multipolar radiofrequency ablation leads to incomplete ablation ex vivo. International journal of hyperthermia: the official journal of European Society for Hyperthermic Oncology, North American Hyperthermia Group. 2016;32(7):749–56. 10.1080/02656736.2016.1196395 . 27400818

[15] Guo W, He X, Li Z, Li Y. Combination of Transarterial Chemoembolization (TACE) and Radiofrequency Ablation (RFA) vs. Surgical Resection (SR) on Survival Outcome of Early Hepatocellular Carcinoma: A Meta-Analysis. Hepato-gastroenterology. 2015;62(139):710–4. . 26897959

[16] Cardella JF, Kundu S, Miller DL, Millward SF, Sacks D, Society of Interventional R. Society of Interventional Radiology clinical practice guidelines. Journal of vascular and interventional radiology: JVIR. 2009;20(7 Suppl):S189–91. 10.1016/j.jvir.2009.04.035 . 19559998

[17] Lencioni R, Llovet JM. Modified RECIST (mRECIST) assessment for hepatocellular carcinoma. Seminars in liver disease. 2010;30(1):52–60. 10.1055/s-0030-1247132 . 20175033 PMC12268942

[18] Kashima Y, Miyazaki M, Ito H, Kaiho T, Nakagawa K, Ambiru S, et al Effective hepatic artery chemoembolization for advanced hepatocellular carcinoma with extensive tumour thrombus through the hepatic vein. Journal of gastroenterology and hepatology. 1999;14(9):922–7. . 10535476 10.1046/j.1440-1746.1999.01966.x

[19] Itoh A, Sadamori H, Yabushita K, Monden K, Tatsukawa M, Hioki M, et al Advanced hepatocellular carcinoma with hepatic vein tumor thrombosis and renal dysfunction after hepatic arterial infusion chemotherapy effectively treated by liver resection with active veno-venous bypass: report of a case. BMC cancer. 2016;16:705 10.1186/s12885-016-2749-4 ; PubMed Central PMCID: PMC5009678. 27586890 PMC5009678

[20] Zhang T, Huang JW, Bai YN, Wu H, Zeng Y. Recurrence and survivals following hepatic resection for hepatocellular carcinoma with major portal/hepatic vein tumor thrombus. Hepatology research: the official journal of the Japan Society of Hepatology. 2014;44(7):761–8. 10.1111/hepr.12185 . 23763458

[21] Shaohua L, Qiaoxuan W, Peng S, Qing L, Zhongyuan Y, Ming S, et al Surgical Strategy for Hepatocellular Carcinoma Patients with Portal/Hepatic Vein Tumor Thrombosis. PloS one. 2015;10(6):e0130021 10.1371/journal.pone.0130021 ; PubMed Central PMCID: PMC4468137. 26076461 PMC4468137

[22] Wright AS, Sampson LA, Warner TF, Mahvi DM, Lee FT Jr. Radiofrequency versus microwave ablation in a hepatic porcine model. Radiology. 2005;236(1):132–9. 10.1148/radiol.2361031249 . 15987969

[23] Brace CL, Laeseke PF, Sampson LA, Frey TM, van der Weide DW, Lee FT Jr. Microwave ablation with a single small-gauge triaxial antenna: in vivo porcine liver model. Radiology. 2007;242(2):435–40. 10.1148/radiol.2422051411 ; PubMed Central PMCID: PMC1945245. 17255414 PMC1945245

