# Correction: Percutaneous thermal ablation combined with TACE versus TACE monotherapy in the treatment for liver cancer with hepatic vein tumor thrombus: A retrospective study

**DOI:** 10.1371/journal.pone.0204535

**Published:** 2018-09-18

**Authors:** Yang Wang, Liang Ma, Zhuhui Yuan, Jiasheng Zheng, Wei Li

There is an error in the affiliation for author Wei Li. The affiliation should be:

Center of Interventional Oncology and Liver Diseases, Beijing You’an Hospital, Capital Medical University, Beijing, China.
