# Supplementary material for: Percutaneous thermal ablation combined with TACE versus TACE monotherapy in the treatment for liver cancer with hepatic vein tumor thrombus: A retrospective study
Source: PLoS One. 2018 Jul 31;13(7):e0201525. doi: 10.1371/journal.pone.0201525 (PMC6067729; doi:10.1371/journal.pone.0201525)
Supplement: S1 Table — (DOCX) [file pone.0201525.s001.docx]

**Table S1.** Details of HVTT ablation procedures

| Case | Tumor type | HVTT location | Lenth of TT, mm | Technique | Ablation system | Ablation details |
| --- | --- | --- | --- | --- | --- | --- |
| 1 | HCC | Right HV,  Within TB | 27 | MWA | Qinghai Ltd.,Nanjing, P.R.China | 60-70W, 3-6min |
| 2 | HCC | Right HV,  Beyond TB | 70 | MWA | Qinghai Ltd.,Nanjing, P.R.China | 60W, 6min |
| 3 | HCC | Right HV,  Beyond TB | 30 | RFA | RITA Medical Systems, Mountain View, CA | 95℃, 20min |
| 4 | HCC | Middle HV,  Beyond TB | 43 | RFA | Valleylab, ACTC1525, Boulder, Covidien | 15min |
| 5 | HCC | Right HV,  Beyond TB | 43 | RFA | Valleylab, ACTC1525, Boulder, Covidien | 12min |
| 6 | HCC | Left HV,  Beyond TB | 31 | RFA | VIVA RF system, STARmed, Goyang, Korea | 70-100W, 6-12min |
| 7 | HCC | Middle HV,  Beyond TB | 66 | RFA | RITA Medical Systems, Mountain View, CA | 90℃, 15min |
| 8 | HCC | Middle HV,  Beyond TB | 65 | MWA | Qinghai Ltd.,Nanjing, P.R.China | 30W-40W, 5min |
| 9 | HCC | Left HV,  Beyond TB | 35 | MWA | Qinghai Ltd.,Nanjing, P.R.China | 60W, 6min |
| 10 | HCC | Middle HV, Within TB | 36 | MWA | Qinghai Ltd.,Nanjing, P.R.China | 30-60W, 4-8min |
| 11 | HCC | Middle HV,  Beyond TB | 40 | MWA | Qinghai Ltd.,Nanjing, P.R.China | 60W, 6min |
| 12 | HCC | Accessory HV,  Beyond TB | 85 | MWA | Qinghai Ltd.,Nanjing, P.R.China | 60-70W, 6min |
| 13 | ICC | Left+middle HV,  Beyond TB | 65 | MWA | Qinghai Ltd.,Nanjing, P.R.China | 50-100W, 3-10min |

Abbreviations:HVTT, hepatic vein tumor thrombus; TT, tumor thrombus; HCC, hepatocellular carcinoma; HV, hepatic vein; TB, tumor boundary; MWA, microwave ablation; RFA, radiofrequency ablation; ICC, intrahepatic cholangiocarcinom.
