# Supplementary material for: Percutaneous thermal ablation combined with TACE versus TACE monotherapy in the treatment for liver cancer with hepatic vein tumor thrombus: A retrospective study
Source: PLoS One. 2018 Jul 31;13(7):e0201525. doi: 10.1371/journal.pone.0201525 (PMC6067729; doi:10.1371/journal.pone.0201525)
Supplement: S2 Table — (DOCX) [file pone.0201525.s002.docx]

**Table S2. Essentials for the ablation of HVTT**

| No. of essentials | Content |
| --- | --- |
| 1 | If the HVTT was within the tumor boundary, it could be ablated with the tumor tissue en bloc. |
| 2 | If the HVTT was beyond the tumor boundary, there are several essentials for the ablation procedure as follows, according to our experience. |
| 2a | Pre-operative contrast-enhanced CT should be obtained to help design the route of insertion. |
| 2b | Ablating the hepatic thrombus could be more difficult and risky than ablating the tumor, and the thrombus is recommended to be ablated prior to the tumor lesion. |
| 2c | The apex part (closer to the second hepatic hilum) of HVTT should be ablated first, then the connecting part and basal part (closer to the originating tumor) of HVTT, respectively. |
| 2d | To reach the HVTT, the optimal puncture path is through the hepatic parenchyma, without involving important vessels and biliary ducts. |
| 2e | If tumor nodule is on the puncture path, it should be ablated while advancing the needle, and the needle track should also be ablated while withdrawing the needle. Thust needle track dissemination could be avoided. |
